# Insights From Analysis of Human Antigen-Specific Memory B Cell Repertoires

**DOI:** 10.3389/fimmu.2018.03064

**Published:** 2019-01-15

**Authors:** Hemangi B. Shah, Kenneth Smith, Jonathan D. Wren, Carol F. Webb, Jimmy D. Ballard, Rebecka L. Bourn, Judith A. James, Mark L. Lang

**Affiliations:** ^1^Department of Microbiology and Immunology, University of Oklahoma Health Sciences Center, Oklahoma City, OK, United States; ^2^Arthritis and Clinical Immunology, Oklahoma Medical Research Foundation, Oklahoma City, OK, United States; ^3^Department of Biochemistry and Molecular Biology and Geriatric Medicine, University of Oklahoma Health Sciences Center, Oklahoma City, OK, United States; ^4^Division of Rheumatology, Immunology and Allergy, Department of Cell Biology and Internal Medicine, University of Oklahoma Health Sciences Center, Oklahoma City, OK, United States; ^5^Department of Medicine and Pathology, University of Oklahoma Health Sciences Center, Oklahoma City, OK, United States

**Keywords:** memory B cells, vaccination, monoclonal antibody, antibody repertoires, next generation sequencing

## Abstract

Memory B cells that are generated during an infection or following vaccination act as sentinels to guard against future infections. Upon repeat antigen exposure memory B cells differentiate into new antibody-secreting plasma cells to provide rapid and sustained protection. Some pathogens evade or suppress the humoral immune system, or induce memory B cells with a diminished ability to differentiate into new plasma cells. This leaves the host vulnerable to chronic or recurrent infections. Single cell approaches coupled with next generation antibody gene sequencing facilitate a detailed analysis of the pathogen-specific memory B cell repertoire. Monoclonal antibodies that are generated from antibody gene sequences allow a functional analysis of the repertoire. This review discusses what has been learned thus far from analysis of diverse pathogen-specific memory B cell compartments and describes major differences in their repertoires. Such information may illuminate ways to advance the goal of improving vaccine and therapeutic antibody design.

## Introduction

The long-term efficacy of vaccines is determined in large part by the generation of B and T cell memory (1, 2). Memory B cells (Bmem) defend against previously experienced pathogens by differentiation into antibody (Ab)-secreting plasma cells (PCs) (3, 4). However, certain pathogens drive functional changes in the Bmem compartment that may be age-dependent and contribute to chronic or recurrent infections (5, 6). Understanding the characteristics and the diversity of protective, ineffective, and pathogenic Bmem responses is likely to aid in the development of efficacious vaccines and therapeutic Abs. In this review we present an overview of Bmem cellular subsets in humans. We highlight recent methods that have allowed us to explore the Bmem Ab gene repertoire. We then examine what is known about the human Bmem Ab repertoires that have been observed following vaccination or infection.

## Memory B Cell Generation

**Figure 1:**
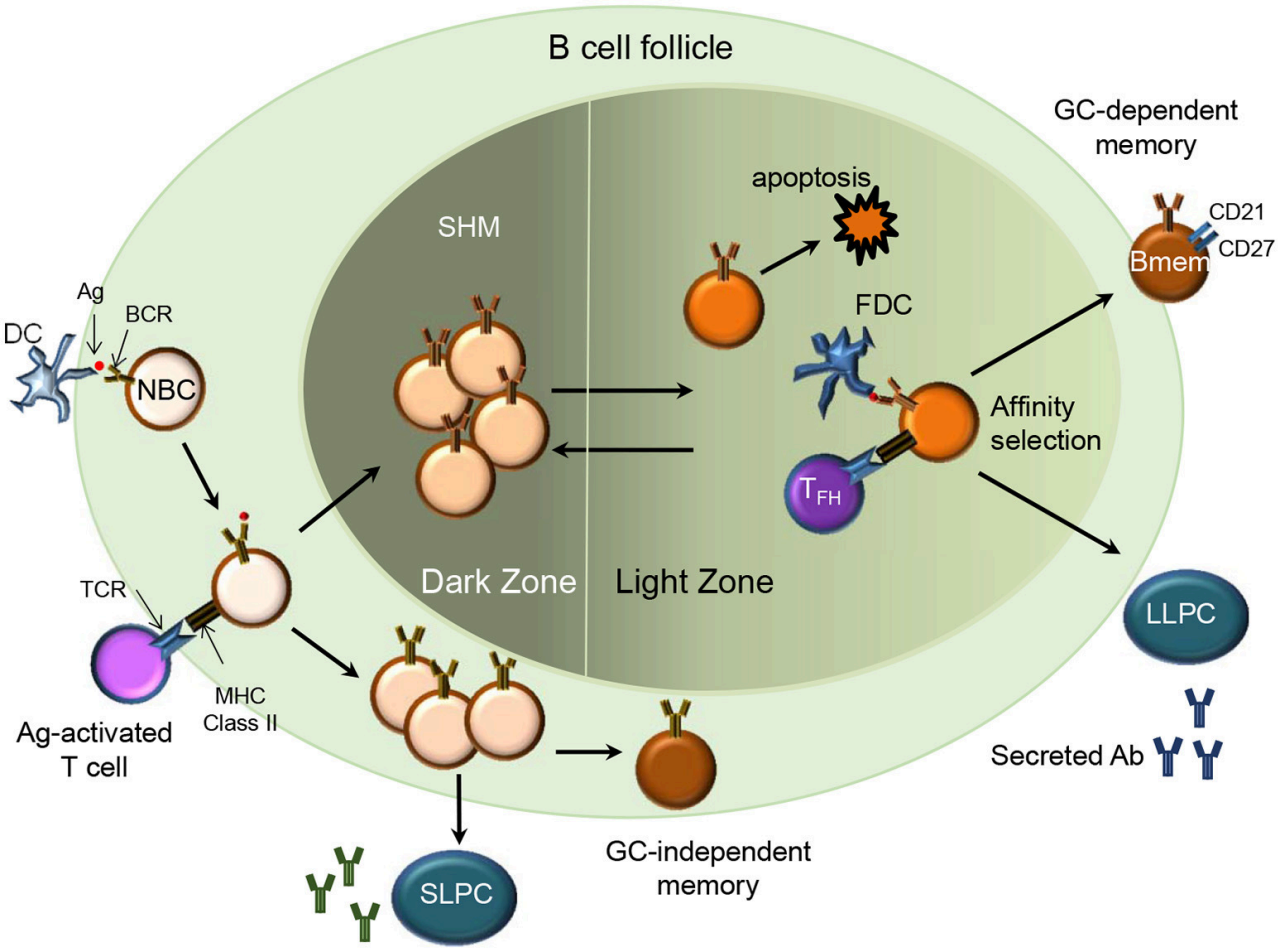
Memory B cell generation (T cell-dependent). In a secondary lymphoid organ, Ag-activated naïve B cells (NBC) via their MHC class II receptors and T cells via their T cell receptors (TCR) form stable interactions upon migrating to the borders of the B cell follicle and T cell zone respectively. B cells then proliferate and form the germinal center (GC)-dependent Bmem, GC-independent Bmem or differentiate into Ab-secreting short-lived plasma cells (SLPC) in an extra-follicular foci. The Ag-specific B cells undergo clonal expansion and SHM in the dark zone. B cells with diversified BCR may relocate to the light zone where they encounter immune complex-coated follicular dendritic cells (FDC) and Ag-specific follicular helper T cells (TFH). Through the process of affinity selection, the B cells with high affinity for the Ag survive while those with low affinity undergo apoptosis. After affinity maturation, the B cells can either re-enter the GC or exit the GC as Bmem or Ab-secreting long-lived plasma cells (LLPC) that home to their survival niche in the bone marrow.

B cell receptor (BCR) diversity within the naïve B cell compartment results from the recombination of the variable (V), diversity (D), and joining (J) genes in the heavy (H) chain and VJ genes in the light (L) chain (kappa and lambda) during B cell maturation. During an infection, naïve B cells are exposed to antigen (Ag) in the secondary lymphoid organs and undergo activation and differentiation including somatic hypermutation (SHM) and immunoglobulin (Ig) class switching to produce high affinity Abs. Ag-activated B cells may undergo differentiation into Bmem or short and long-lived PCs. The differentiation into Bmem may occur with or without T cell help and in a germinal center (GC)-dependent or independent manner (7) (Figure 1). This results in Bmem subsets that differ in their effector function and overall capacity for protection (3, 8).

### T-Dependent Bmem

Ag-activated B cells receiving T cell help may differentiate into extrafollicular short-lived PCs, GC-dependent Bmem or GC-independent Bmem (7) (Figure 1). Imaging studies in mice indicate that durable interactions of B cells with cognate follicular helper T cells (Tfh cells) at the B cell- T cell border in spleen and lymph nodes provides T cell help and promotes entry of the B cells in to the GC (9, 10). GC B cells undergo affinity maturation and SHM and upon cognate interaction with GC Th cells differentiate into Bmem or long-lived PCs (11). Mechanisms regulating the differentiation of GC B cells to Bmem are not yet well understood. While most Bmem result from a GC reaction, mouse studies indicate that CD40 signaling via T cells can drive differentiation of GC-independent Bmem (12) and that these cells express Abs with fewer mutations and low affinity (13). However, no clear evidence from human studies supports a GC-independent path for the generation of human Bmem.

### T-Independent Bmem

T-independent Ags such as polysaccharides and other molecules displaying repeating epitopes have long been considered incapable of generating a “memory” response. However, based on murine studies, B-1b cells found predominantly in the peritoneal cavity and marginal zone B cells are the primary precursors for T-independent memory (14). In humans, IgM^+^/IgD^+^/CD27^+^ B cells observed in human peripheral blood and spleen are the only known B cell subset associated with an Ab response to a T-independent Ag (15). Several studies including one involving immunization with a polysaccharide vaccine, demonstrated that the IgM^+^ Bmem in the periphery were derived from splenic marginal zone B cells with a pre-diversified Ig repertoire (15–19).

## Memory B Cell Subsets

While early studies on Bmem in humans and mice predominantly involved IgG^+^ cells, later studies revealed the existence of IgM^+^ Bmem. The use of CD27 as a Bmem surface marker in humans further distinguished the memory from naïve IgM^+^ B cells. These surface markers help identify the potential origin and function of each Bmem subset. A detailed discussion of human Bmem generation, subsets and function has been provided in other reviews (1, 8). Here, we highlight the human Bmem subsets based on their Ig isotypes and their importance during repertoire analysis.

### IgM^+^

The human Bmem compartment was believed to be exclusively or mostly composed of class-switched B cells, however studies have demonstrated the presence of somatically mutated IgM^+^ B cells (20). B cells expressing CD27 and either IgM alone or IgM and IgD represent the IgM^+^ Bmem subset. While the GC-dependence of IgM only Bmem is accepted, the origin of the IgM^+^/IgD^+^ subset is contentious. In their review of human Bmem, Seifert and Küppers (8) discuss evidence for a GC origin of this IgD^+^ subset including: (a) their ability to reenter the GC upon secondary challenge, (b) their tendency to differentiate to PCs, (c) their longevity, and (d) their transcriptome profile. Thus, the GC-dependent origin of IgM^+^/IgD^+^ cells supports their characterization as Bmem.

The peripheral blood IgM^+^/IgD^+^/CD27^+^ Bmem correspond to circulating splenic marginal zone B cells and contribute to protection against blood-borne T-independent pathogens (15, 21). Pneumococcal polysaccharide-specific IgM^+^/IgD^+^/CD27^+^ Bmem displayed somatic mutations when isolated post immunization with a T-independent vaccine (Pneumovax) (22). A vesicular stomatitis virus-Ebola vaccine generated a neutralizing IgM response that persisted despite administration of a booster vaccine (23). These studies point to a previously under-appreciated contribution of hyper-mutated IgM^+^ Bmem to protection against pathogens.

### IgG^+^

IgG-expressing Bmem constitute 15–20% of the peripheral blood B lineage cells in adults. The structure and composition of the Ag as well as regulatory factors determine how the IgG^+^ Bmem cells class switch to express mainly IgG1, IgG2, or IgG3 and to a lesser extent IgG4. Some examples include: the importance of the polysaccharide-specific IgG2 response in protection against *Streptococcus pneumoniae*; the predominance of IgG1 and IgG3 subclasses generated against HIV, Ebola virus, and *Plasmodium falciparum;* and IgG4 responses to *Schistosoma mansoni* (24). However, not all the subclasses expressed are equally efficacious. For example, IgG3 was found to be more effective at neutralizing HIV than IgG1 (25). While the majority of the IgG expressing Bmem are CD27^+^, 20–25% lack CD27 expression (26). IgG^+^/CD27^−^ Bmem cells have fewer mutations in their V regions and predominantly express the IgG3 subclass (26, 27). This subpopulation is increased in the elderly and is hypothesized to represent an “exhausted” Bmem pool (28). IgG^+^ Bmem upon reactivation typically differentiate into PCs rather than re-enter the GC. Therefore, the IgG subclass is also an important aspect of the Ab repertoire that should be considered in analyses of data sets.

### IgA^+^

IgA-expressing Bmem are associated with mucosal immune responses and tend to arise from and localize in the intestine and mucosa-associated lymphoid tissue. They make up ~10% of the B cells in the periphery. While most IgA^+^ Bmem are CD27^+^, there is evidence of less mutated IgA^+^ CD27^−^ cells undergoing low levels of proliferation and expressing poly-reactive Abs (29, 30). This phenotype is indicative of cells generated independent of the GC. Alternatively, an early exit from the GC allows for a broader and less mutated IgA^+^ Bmem which could cross-protect against related pathogens such as enterotoxigenic *E. coli* and *Vibrio cholera* (31). A recent study demonstrated that IgM^+^ Bmem shared gut-specific gene signatures with IgA^+^ Bmem, were related to some IgA^+^ clonotypes and could switch to IgA upon T-dependent or independent signals (32). Sustained Ag presence could drive a protective IgA response and could be utilized to improve oral vaccines.

### IgE^+^

Although the presence of IgE antibodies and their causal relationship with atopic diseases such as allergy and asthma is well established, their generation is not well understood and they are detected at very low levels in human peripheral blood. Studies in mouse models have demonstrated the potential for “sequential switching” wherein IgG1 cells switch to IgE Ab-secreting cells (33–35). Another study examined the repertoire of human parental Bmem and their progenies. In that study, it was demonstrated that high affinity IgE-secreting PC clones were derived from the selection and expansion of rare high affinity IgG1 Bmem clones without undergoing further mutation (36). Antibody repertoire analysis of IgE^+^ B cells in patients with seasonal rhinitis demonstrated that the V gene usage was limited and similar across multiple patients (37). Furthermore, people with parasitic infections and patients with atopic dermatitis had less clonal diversity and lower frequency of SHM in their IgE repertoires than those with asthma (38). These differences reiterate the importance of examining the pathogen-directed IgE repertoire in the context of specific pathological events.

### Atypical, Tissue-Like, or Exhausted Memory B Cells

**Figure 2:**
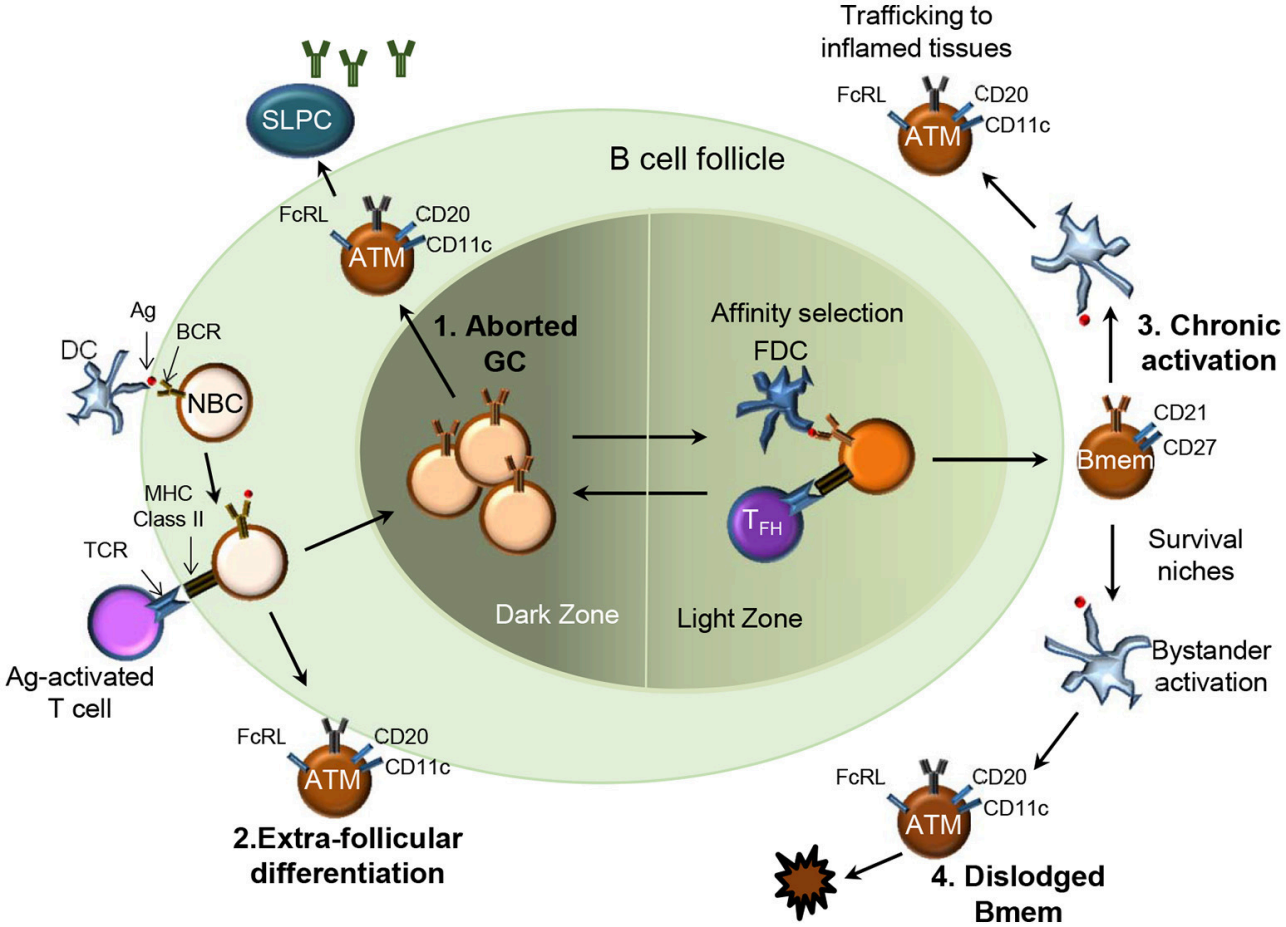
Four potential pathways for generation of atypical memory B cells in chronic infections. Several pathways for the origin of atypical Bmem (ATM) have been proposed: 1.They may be Bmem derived from prematurely aborted GCs (top left), 2. Bmem derived from an extra-follicular differentiation pathway (bottom left); 3. Chronic Ag-mediated activation of previously functional Bmem may drive the expansion of ATM (top right), or 4. Represent the end stage for Bmem dislodged from survival niches due to repeated bystander activation (bottom right).

HIV, *Mycobacterium tuberculosis, Plasmodium falciparum*, and *Hepatitis C virus* cause chronic infections and account for more than five million deaths a year. The chronic presence of Ag, prematurely aborted GC, extra-follicular differentiation or loss of survival niche may drive the expansion of a phenotypically and functionally altered Bmem subset referred to as “exhausted,” “tissue-like,” or “atypical” Bmem (Figure 2) (39–42). Distinct from typical CD27^+^ Bmem, these atypical Bmem do not express CD27 and cannot be stimulated via their BCR to subsequently produce Ab. HIV-associated CD21^lo^/CD27^−^ cells expressed high levels of CD20 and their expression of CD11c, T-bet and inhibitory receptors of the Fc receptor like (FcRL) family distinguished them from other B cell subsets (40). Their resemblance to the FcRL4-expressing cells resident in the tonsils defined them as “tissue-like” Bmem. The tonsillar CD20^hi^/CD21^lo^/CD27^−^/FcRL4^+^ B cells had undergone isotype switching and SHM similar to CD27^+^ Bmem but were non-responsive to stimulation through BCR cross-linking (43). Atypical FcRL4-expressing Bmem were also observed to be increased in frequency in individuals with chronic HCV infection (44) and in those with active and latent TB infection (45). A similarly expanded subset of atypical Bmem was observed in those repeatedly infected with *P. falciparum* (46, 47). The atypical Bmem in patients with malaria express FcRL5 rather than the FcRL4 expression observed on tissue-like Bmem in HIV (48). In keeping with the “exhausted” phenotype, these FcRL5^+^ atypical cells were more refractive to BCR crosslinking and CpG stimulation as compared to FcRL5^−^ Bmem. While T-bet-expressing, CD21^low/−^ B cells have been observed in individuals with autoimmune conditions such as rheumatoid arthritis (49) and systemic lupus erythematosus (50), they likely differ phenotypically and functionally from cells arising during chronic infections (6). Chronic immune stimulation and inflammation (Figure 2) are thought to contribute to the expansion of atypical Bmem that are unable to secrete Ab which could explain lack of acquisition of immunity against chronic infections.

## Human Monoclonal Antibody Production Methods to Sample the Memory B Cell Repertoire

**Figure 3:**
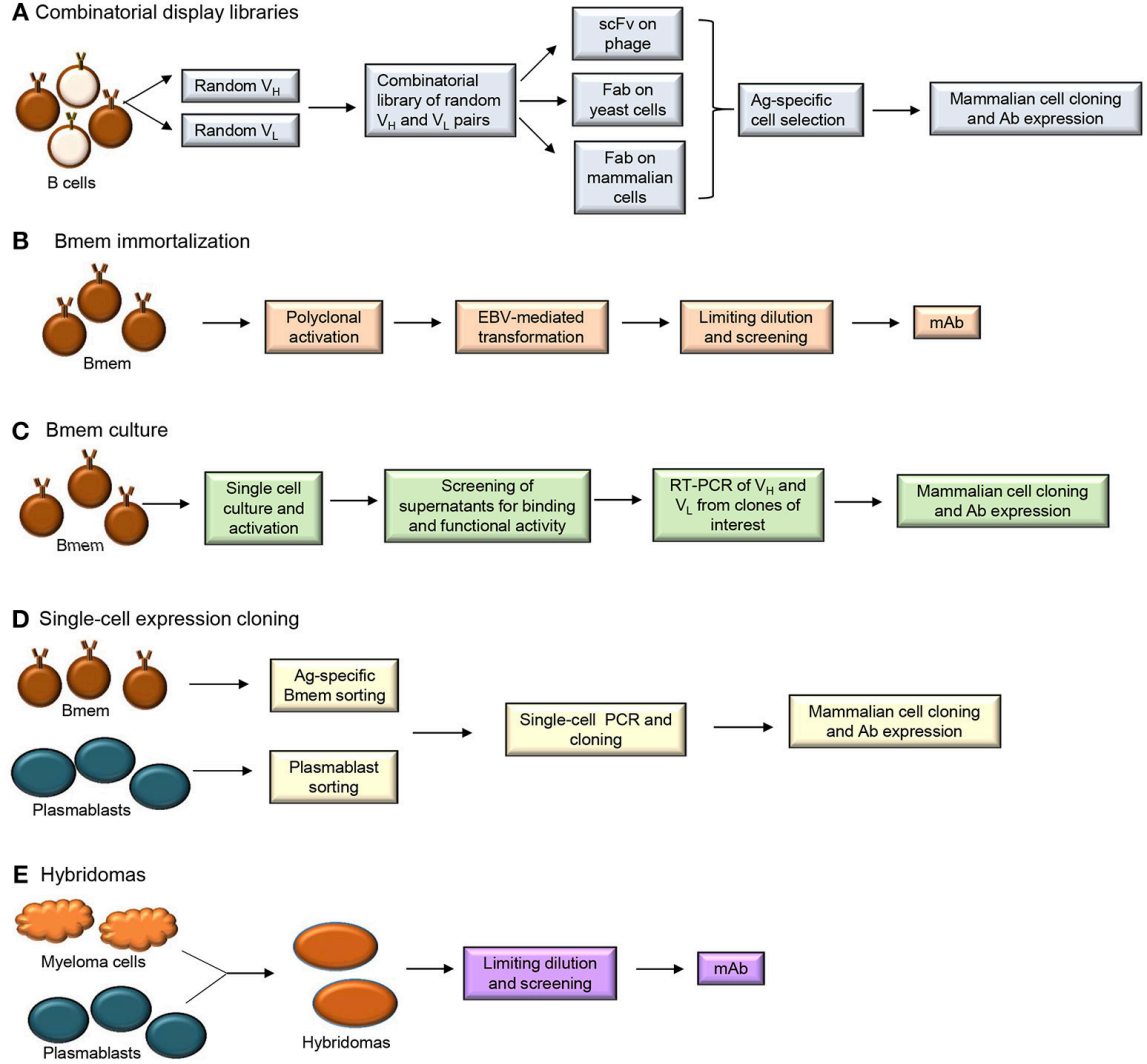
Human monoclonal antibody isolation methods **(A)** Combinatorial display libraries **(B)** Bmem immortalization **(C)** Bmem cell culture **(D)** Single-cell expression cloning and **(E)** Hybridoma approaches.

Methods used to produce human monoclonal Abs (mAbs) include combinatorial display libraries, B cell immortalization, single-cell expression cloning and hybridoma generation (Figure 3) (51–53). Each of these methods has been used to improve our understanding of the Bmem repertoire.

Generation of combinatorial display libraries of single-chain variable fragments (scFvs) and Ag-binding fragments (Fabs) (Figure 3A) is a high-throughput screening method that has allowed discovery of several pathogen-neutralizing Abs (54–57). Libraries of Ab V genes are generated from B cells isolated from an individual post-immunization or post-infection. These random pairs of H and L chain genes are then expressed on phage, yeast or mammalian cells. The Ag-specific Ab-expressing cells or particles are selected and cloned into mammalian cells for mAb expression. Since the H and L chain pairing occurs randomly during this process, the resulting Ab does not represent a pairing that may occur naturally in the immune system. This technique can allow generation of higher affinity Abs but does not allow characterization of the properly-paired Ag-specific B cell repertoire.

B cell immortalization (Figure 3B) involves transformation of Bmem using Epstein Barr Virus (EBV) in the presence of TLR (Toll-like receptor) 9 agonist CpG DNA (58). Total blood lymphocytes or isolated IgG^+^ Bmem are typically cultured with EBV and CpG and/or additional co-stimulants for 1–2 weeks to allow proliferation of B cells and secretion of Abs. Supernatants from the cultures are then harvested to test for Ag-specific Ab after which the individual Ig H and L chain pairs are cloned and sequenced. EBV-transformed B cells allow Bmem analysis but require screening of significant numbers of cells (~1,000) to identify the very few Bmem (~10) of interest. Bmem can also be immortalized by forced expression of BCL6 and BCL-XL and when cultured in the presence of CD40L/IL-21 these plasmablast-like transduced cells secrete Ab in the supernatant (59). While plasmablasts are not amenable to immortalization, recent studies have demonstrated that both Bmem and plasmablasts can be cultured *in vitro* (Figure 3C), without immortalization, for Ab production (60–62).

Single-cell expression cloning involves application of reverse transcription-polymerase chain reaction (RT-PCR) at the single-cell level for the amplification of Ig H and L chain genes that can be cloned and expressed in mammalian cell lines (Figure 3D). Plasmablasts and Bmem have been sorted from individuals post-vaccination or post-infection to generate human mAbs by single-cell expression cloning. This technique involves specific PCR amplification of Ab transcripts from individual cells and is technically challenging and dependent upon high quality primers (63). Application of single-cell expression cloning for repertoire analyses can be difficult in peripheral B cell subsets that have fewer Ig transcripts per cell than plasmablasts (64). Plasmablasts that secrete larger quantities of Ig are more amenable to these types of protocols. Although number of circulating plasmablasts are typically low in healthy individuals, 5–8 days after vaccination (or infection) there is a transient but large population of Ag-specific Ig-secreting cells that can be easily isolated (63, 65). This population can be distinguished from the IgG^+^ Bmem population which peaks at 2–3 weeks post-immunization. Plasmablasts sorted from human PBMC 1 week after vaccination against influenza, *Bacillus anthracis* and *Streptococcus pneumoniae* have successfully yielded Ag-specific and high affinity mAbs (52).

Ag-specific plasmablasts and Bmem have been isolated by fluorescence-activated cell sorting (FACS) using reagents that bind Ag-specific cells (Ag baiting) (66–71). IgG^+^ PCs and plasmablasts are difficult to isolate in an Ag-specific manner due to their limited surface expression of Ig (72). However, a flow cytometry-based Ig capture assay was established to sort IgG^+^ Ag-specific plasmablasts for three vaccine Ags, namely HIV gp140, tetanus toxin and Hepatitis B surface Ag (73). This method captured the secreted Ab from the plasmablast enabling the fluorescent Ag probe to identify Ag-specific cells. Isolating Ag-specific human Bmem is also challenging since they circulate at very low frequencies in the periphery (74) but they can be expanded *in vitro* and immortalized using EBV-transformation techniques mentioned earlier. A recent study isolated human CD27^+^ Bmem and demonstrated that “an *in vitro* booster vaccination” consisting of streptavidin-coated nanoparticles conjugated with biotinylated Ag and CpG DNA generated Ag-specific mAbs in a period of 6 days (75).

Traditional mAb generation methods such as hybridoma technology (Figure 3E) involve the production of Ab from a hybridoma clone formed by the fusion of a myeloma cell with B cell from a donor or an immunized animal (53, 76). Although this method can generate high affinity mAb, it has to be combined with transgenic humanized mouse strains to obtain fully human mAb (77).

## Next Generation Sequencing to Study Memory B Cell Repertoire

The ability to accurately perform deep sequencing of Ab VDJ regions has provided valuable insights into the regulation and evolution of the Bmem repertoire. Rapidly developing high-throughput next-generation sequencing technologies have achieved high resolution in the number of single chains that can be sequenced in one experiment (78). However, information about the endogenous pairing of IgH/IgL genes cannot be obtained by single chain sequencing which then hinders an accurate representation of the B cell repertoire. This suggests that improved methods for template preparation, sequencing and imaging, and data analysis are required for more accurate and efficient large scale analysis of functional Ab repertoires (79).

A recently developed “ultra high-throughput” method amplifies IgH/IgL genes from a B cell captured within a droplet to create a single DNA amplicon allowing sequencing of paired chains (80). In addition, simultaneous sequencing of barcoded Ig genes along with co-expressed functional genes will better represent the Ab repertoire (79). Combining large datasets from several reads (deep sequencing) of B cell Ab repertoire sequences (BCR/Rep-seq) with data from mass spectrometry of serum Ab (Ig-seq) will allow comparison of the Ab repertoires in the two compartments (81, 82).

Single-cell RNA-seq technologies typically generate large datasets that require bioinformatics tools and expertise for deconvolution and interpretation. Several “pipelines” have been developed for BCR sequence analysis and will likely continue to evolve to accommodate data complexity (83, 84). As the cost per kilobase of high-throughput sequencing (HTS) has dropped, the past 3 years alone has seen a rapid increase in the number of published resources for analysis of B-cell sequence data (Table 1).

**Table 1:** Bioinformatics resources for analysis and comparison of B-cell antibody sequence diversity.

**Resource name**	**Type**	**Description/purpose**
B-cell sequences (85)	Database	>37 M B-cell sequence reads from 3 individuals plus analysis tools
SONAR (86)	Software	B-cell repertoire and lineage analysis
IMPre (87)	Software	Predict T/B-cell germline sequence using HTS data
BASIC (88)	Software	B-cell receptor assembly using single-cell RNA-seq data
VDJServer (89)	Software	Tools for B-cell sequencing and rearrangements
sciReptor (90)	Software	Single-cell Ig repertoire analysis
ClonoCalc/Plot (91)	Software	Generate figures to summarize clonal diversity/expansion
IRProfiler (92)	Software	Immune receptor profiling from HTS
ImmunediveRsity(93)	Software	Analyze B-cell repertoire diversity from sequencing data
Repertoire analysis (94)	Review	Software and methods for B-cell repertoire analysis

*HTS, High-Throughput Sequencing*.

As we will discuss, the techniques outlined above have been applied to Bmem subsets, allowing an improved understanding of their development and differentiation during specific diseases and post-vaccination.

## Infection-Induced Memory B Cell Antibody Repertoires

While there has been significant research on mAbs as therapeutics for multi-drug resistant Gram negative bacteria [reviewed in (95)], our knowledge on how they shape human Bmem repertoires is lacking and deserves further attention. The reader is also referred to three studies on the Bmem repertoire against *Vibrio cholerae, Klebsiella pneumoniae*, and *Hemophilus influenzae* (96–98). In this article, we have focused on Gram positive infections for which a larger number of published studies was available for analysis.

## Gram Positive Bacterial Infections

### Bacillus anthracis

*Bacillus anthracis* is a gram positive, rod-shaped bacteria that causes a serious, often fatal, infection in humans. Alarmingly, *B. anthracis* has been used as a biological weapon highlighting the seriousness of anthrax disease. Antibiotics are used for anthrax treatment and as post-exposure prophylaxis. The use of FDA approved Anthrax Vaccine adsorbed (AVA) is restricted to military personnel and those who might face occupational anthrax exposure. AVA is predominantly composed of protective Ag (PA), which is a component of the tripartite anthrax exotoxin. AVA elicits toxin neutralizing Abs that are protective, but generating the protective response requires multiple vaccinations and ongoing annual boosters (99). The vaccine generates a protective short-lived anti-PA IgG response in humans, but both human and animal studies using AVA demonstrated a long-lived anti-PA Bmem response (100, 101). Using mAbs from PA-specific B cells isolated from seven AVA-vaccinated individuals, Reason and coworkers showed that although a majority of the mAbs bound a specific 20 kDa region on the PA monomer, their VH region sequence analysis revealed 64 unique gene rearrangements (102). Ab-secreting cells were also isolated from individuals 7 days following AVA or recombinant PA vaccination to generate and characterize human PA-specific mAbs (103, 104). Several of those mAbs demonstrated *in vitro* toxin neutralization and were protective *in vivo* following challenge of mice with bio-active anthrax toxin. While several PA-specific mAbs are in development, the recombinant human mAb raxibacumab is the only one approved for use in treatment of inhalational anthrax (105).

### Clostridium tetani

*Clostridium tetani* secretes tetanus toxin, which is responsible for the symptoms associated with tetanus disease. Since the introduction of tetanus toxoid (TT)-containing vaccines in the mid-1940s, the incidence of reported tetanus cases in the United States declined by over 98% from 0.39 per 100,000 population in 1947 to 0.01 per 100,000 population in 2016 (106). The tetanus-specific Ab repertoire has been characterized by several groups using different methods. Analysis of Fab libraries created from plasmablasts isolated day 6 post-TT vaccination indicated that 100 B cell clones and their hyper-mutated variants comprised the human polyclonal Ab repertoire (107). The follow-up study examined the plasmablast V gene region after 3 TT vaccinations and suggested that the majority of the B cell clones developed in response to a single vaccination event (108). Libraries created using a single human VL region paired with a collection of VH regions resulted in diverse and high affinity Abs where the TT-specificity was encoded solely by VH (109). Lavinder and coworkers used high-resolution liquid chromatography tandem MS proteomic analyses of serum Abs coupled with next-generation sequencing of the V gene repertoire in peripheral B cells to understand the serum IgG and B cell repertoire following TT booster vaccination (110). This study confirmed the previous finding that TT^+^ serum IgG comprised ~100 clonotypes, but they found that only 3 clonotypes accounted for >40% of the response and that <5% of plasmablast clonotypes account for the Abs detected in the serum 9 months post-vaccination. As TT vaccines are very effective in preventing tetanus, characterizing the Bmem repertoire generated in response to this vaccine will provide information that is valuable in the improvement of vaccines to other pathogens.

### Streptococcus pneumoniae

*Streptococcus pneumoniae* is a causative agent for community-acquired pneumonia, a health care burden worsened by antibiotic resistance. Despite effective vaccines, the lack of adequate coverage especially in adults results in a large at-risk population (111). This problem is compounded by some serotypes not being represented in current vaccines (112).

The Ab repertoire in response to vaccination with the 23-valent polysaccharide vaccine and the conjugate vaccine has been examined (113–116). Full-length fully human serotype-specific mAbs and anti-cell wall polysaccharide mAbs generated from individuals 7 days post-vaccination were examined for their V gene usage and clonal families (113). Zhou and colleagues generated human Fab fragments specific to the capsular polysaccharide of *S. pneumoniae* strains 23F and 6B to determine their H and L chain variable gene usage (115, 116). Bmem-derived human hybridomas were generated from recipients of the conjugate vaccine to study the structure-function correlation of mAbs and how that may affect the B cell repertoire during invasive pneumococcal infection (114). While different methods were utilized to examine the Ab repertoire in the studies cited above, they all demonstrated an increased frequency of SHM in response to primary vaccination. This suggested that the recipients were either asymptomatic carriers or previously exposed to a wild-type strain of *S. pneumoniae* which allowed the vaccination to elicit a memory response. Furthermore, the studies also revealed that the Ab repertoire across individuals was oligoclonal based on their limited usage of VH and VL families and similar H and L pairing for specific serotypes. Two studies identified IgG2 and IgA H chains matching the Ab isotype and response found in the donor sera indicating that the Fabs and Abs derived were from a response to *S. pneumoniae* infection (114, 115). Further characterization of the full-length fully human serotype-specific mAbs revealed a preferential use of lambda over kappa light chains in response to certain serotypes (117).

The emergence of *S. pneumoniae* serotypes not covered in the vaccine has driven the efforts to develop a “universal” vaccine that is not serotype-specific. Pneumococcal surface protein A (PspA) is found in all *S. pneumoniae* isolates and studies examining the Ab response against its proline-rich region have indicated that PspA may be a good vaccine candidate (118–120). A detailed examination of the human anti-PspA Ab repertoire would allow a better understanding of the efficacy of PspA-based vaccines.

## Parasitic Infection

### Plasmodium falciparum

*Plasmodium falciparum* is the mosquito-borne parasite that causes malaria. According to the CDC, malaria caused an estimated 216 million cases of malaria and 445,000 deaths worldwide in 2016. With repeated exposure, older children and adults slowly develop resistance to severe illness and death but never achieve complete resistance to infection (121). In humans, *P. falciparum* infection generates a long-lived atypical Bmem response which develops slowly, after many years of malaria exposure, and is limited in magnitude (122). The neutralizing IgG^+^ Abs produced by atypical Bmem seen during malaria infection in adults differ in their V region repertoire from Abs produced by classical Bmem (123). A similar study that examined the V gene of Abs in children, found no differences between the classical and atypical Bmem repertoire (121). Infant immune repertoires are poorly characterized and thought to be limited in their ability to respond to Ag challenge (124). A recent study isolated Bmem from infants and toddlers pre-infection and during acute malaria infection and examined the Ab repertoire using a method known as molecular identifiers clustering-based immune repertoire sequencing (125). This study found that infant Bmem could acquire over 20 mutations per H chain V region in response to malaria infection and the breadth of repertoire achieved was comparable to that in young adults exposed to malaria. It was also observed that upon malaria re-challenge, Bmem from toddlers previously exposed to malaria could undergo further mutations while retaining IgM expression. The ability to sequence and analyze the immune repertoire even from small samples has allowed a better understanding of the infant Bmem compartment which in turn can inform vaccine design and strategy to prevent malaria in this age group.

## Viral Infections

The role of serum Ab as an immunotherapy in viral infections has been studied for over 50 years and its importance in prophylaxis is well recognized (126). Improved investigative tools have enabled us to identify neutralizing viral epitopes that can influence vaccine design and Ab engineering. Herein, we focus on the B cell and Bmem repertoires of some more commonly studied viruses.

### Influenza Virus

Influenza virus causes annual epidemics and poses a significant risk for mortality in immunocompromised individuals, the elderly (>65 years) and young children (<5 years). Due to its ability to constantly evolve and adapt, a “universal Influenza vaccine” has eluded investigators. The neutralizing Ab response to viral infection or vaccination mainly targets the glycoprotein hemagglutinin (HA) which prevents entry into target cells, while Abs directed against the second glycoprotein neuraminidase (NA) prevent virus exit and spread (52, 127). The Abs targeting the HA globular head are limited in breadth. In an effort to generate broadly neutralizing Abs, the focus has been on targeting the HA stem region which is conserved across strains. Abs neutralizing most strains in H1, H5, and H9 clades have been discovered by several groups using phage display and immortalization of Bmem cells (128–131). High affinity human Abs have been cloned from IgG^+^ Ab-secreting cells 1 week after vaccination. In that study it was observed that the repertoire had limited clonality but an accumulation of somatic mutations within each clone, resulting in extensive intraclonal diversity (63). A recent study examined the HA-specific Bmem repertoire from three unrelated individuals that were known to have BCRs cross-reactive to HA from group 1 and 2 influenza viruses (132). They characterized the clonal lineage and determined that germline receptors had the capacity to recognize epitopes on the head of the HA trimer of divergent Influenza subtypes (H1 and H3 subtype). Furthermore, they showed that the clonally related Abs bound both H1 and H3 HA via their long HCDR3 loop which had the same length and related amino acid sequence. The cross-reactive Abs bound H1 and H3 with differing affinities, suggesting that sequential exposure to each of the Influenza subtypes may have driven affinity maturation of those Abs. Other Ab repertoire analyses have revealed the presence of signature sequences and key somatic mutations in HCDR3 and the preferential VH gene usage for the generation of broadly neutralizing anti-influenza Abs (127, 133).

### Human Immunodeficiency Virus

Human Immunodeficiency Virus infected an estimated 1.1 million people in the United States at the end of 2015 (134). With an error-prone reverse transcriptase and its ability to replicate rapidly, multiple variants of HIV co-exist in the host and allow successful evasion of the innate and adaptive immune response. The exposed HIV envelope glycoproteins (Env) are the most accessible to Ab and HIV has evolved mechanisms to avoid recognition by Ab. Evasion mechanisms employed by HIV include generating amino acid changes in the variable regions of the Env and close spacing of the N-glycans to prevent Ab recognition of Env (135). Chronically HIV-1-infected individuals develop anti-Env broadly neutralizing Abs. These Abs have increased SHM, activation-induced cytidine aminase- mediated insertions, deletions in the H or L chain sequences and long HCDR3s or self-reactivity (135). Env-specific single Bmem were sorted from macaques 14 days after immunization and VDJ sequences were examined to reveal 502 unique clonotypes and more than 600 functional sequences (136). Another study examining the Bmem repertoire generated from a chronically infected individual reported that the response was oligoclonal with about 50 clonotypes observed and that the high levels of SHM observed were not restricted to broadly-neutralizing Abs (137). Atypical or exhausted Bmem identified in chronically HIV-infected individuals were demonstrated to have fewer SHM and lower HIV neutralization capacity than resting Bmem-derived mAbs (138). Bmem immortalization, *in vitro* Bmem differentiation and next-generation sequencing techniques have also been applied to samples obtained from infected donors to generate and examine the Ab repertoire with HIV-neutralizing capacity (52, 139). Detailed information regarding differences in the Ab repertoire from “vaccinated” models and chronically infected individuals has been reviewed elsewhere (135).

### Dengue Virus

Dengue Virus (DENV) is a mosquito-borne virus that infects an estimated 390 million people yearly (140). The traditional understanding of Bmem function is that these cells provide the means to rapidly respond to a pathogen upon repeat exposure and provide protection against disease. In DENV infections, however, where sequential infection with more than one serotype is common, the Abs from a memory response to a primary infection can worsen a secondary infection caused by a heterologous dengue virus (141). Halstead proposed Ab-dependent enhancement (ADE) to explain this severity during secondary infection (142). Cross-reactive non-neutralizing Ab generated from a primary response forms Ag-Ab complexes upon secondary infection with a heterologous DENV serotype. The cross-reactive Ab binds Fc receptors allowing more efficient viral entry and replication in Fc-expressing cells resulting in an exacerbated disease. The role of ADE was underscored by the discovery of non-neutralizing cross-reactive Abs generated by sorting IgG^+^ Bmem from DENV-infected individuals (143).

Acute DENV infection results in a large and rapid virus-specific plasmablast response (144) and mAbs derived from plasmablasts of patients with secondary DENV infection revealed a repertoire that was highly affinity matured and clonally expanded (145). These mAbs also demonstrated cross-reactivity to DENV serotypes in binding and neutralization. Appanna and colleagues sorted plasmablasts and Ag-specific Bmem from patients re-infected with DENV and examined their Ab repertoires to conclude that the plasmablast response represented only a fraction of the Bmem repertoire (146). That study emphasized the need to examine each B lineage compartment for a complete understanding of the humoral response. Recently, a DENV-exposed volunteer was vaccinated with a live attenuated tetravalent vaccine (Butantan-DV) and 15 days later plasmablasts were isolated to generate neutralizing Abs (147). The immunization resulted in neutralizing Abs to the previous exposure with DENV type 3 as well as six other mAbs that neutralized three or more serotypes. Additionally, the DENV surface glycoprotein E, the main target for neutralizing Abs, contains regions that are highly conserved between DENV and the re-emerging Zika virus (ZIKV) which results in immunological cross-reactivity (148). The mAbs generated from plasmablasts of DENV-infected individuals cross-reacted with ZIKV (149, 150) and ZIKV-specific mAbs from Bmem of primary ZIKV-infected individuals with no history of DENV infection cross-reacted with DENV E proteins (151).

## Future Directions: Sequencing Antibody Repertoires to Target Urgent and Emerging Threats

### Urgent Threats

In 2013, CDC designated *Clostridioides difficile (C. difficile;* previously *Clostridium difficile)*, Carbapenem-resistant enterobacteriaceae and antibiotic-resistant *Neisseria gonorrhea* as “urgent threats.” Due to the challenge of antibiotic resistance in these pathogens, there has been an emphasis on generating novel therapeutics (95, 152, 153). However, to the best of our knowledge, the Ab repertoire in response to these infections have yet to be examined.

*Clostridioides difficile*, a spore-forming gram positive bacteria, is the leading cause of nosocomial infections in industrialized nations. With at least 500,000 new cases estimated in the US every year, *C. difficile* infection (CDI) is no longer restricted to health care facilities (154). CDI is a potentially debilitating illness ranging from mild diarrhea to a life-threatening pseudomembranous enterocolitis caused by the two *C. difficile* exotoxins Toxin A (TcdA) and Toxin B (TcdB) (154). The existence of recurrent CDI, suggesting mis-directed memory responses and emergence of new hyper-virulent ribotypes, poses a significant challenge to developing appropriate vaccines and therapeutics (155). Several vaccines and mAbs targeting TcdA and TcdB have been introduced in clinical trials and several more are being developed to address treatment and prevention of infection (152). Bezlotoxumab is a humanized mAb that binds TcdB and has been approved by the US FDA for treating recurrent CDI (156). Targeting the toxins for therapy seems appropriate considering studies have demonstrated anti-toxin circulating Ab and Bmem responses in certain individuals with *C. difficile*-associated diarrhea (157) and Bmem response to C-terminal domain (CTD) fragment of TcdB in those with history of CDI (158).

Results from murine studies indicate that while immunization with CTD from the historical strains such as VPI10463 generates a robust recall response with neutralizing Abs that protect against lethal challenge, the CTD from hyper-virulent strains such as BI/NAP1/027 generates a slow Bmem response with toxin-neutralizing Ab that only delays death post-lethal challenge (158). Interestingly, although the TcdB from the two strains share 92% homology, the anti-CTD Abs generated in immunized animal models do not cross neutralize toxin (159). Furthermore a low serum concentration of anti-TcdA and –TcdB Abs have been linked to recurrence (160) underscoring the need to examine in depth the Ab responses in individuals with history of CDI and compare responses in those that suffer recurrences to those that do not. Specifically understanding the Bmem compartment will provide insights into disease recurrence and support better treatment strategies.

### Emerging Threats

Recent outbreaks of Ebola and Zika virus have accelerated the research and development of treatments to counter these threats (161). These renewed research efforts have led to several therapeutic and vaccine candidates against Ebola (162–164) and flaviviruses such as Zika and West Nile virus (165–167) and provided important information about the immune repertoire of individuals vaccinated against or infected with these viruses (23, 168–170). Chikungunya Virus (CHIKV), a mosquito-borne alpha virus first isolated in Tanzania in 1952, is now emerging as a world-wide threat. There are no licensed vaccines or therapies available currently to prevent or treat CHIKV. While the immune response to CHIKV infection is not fully understood, multiple studies have demonstrated the role of passive immunotherapy in controlling CHIKV infection (171–176).

Although great strides have been made to identify and characterize neutralizing mAbs as therapeutics, these Abs are typically derived from very few individuals. Expanding Ab repertoire studies by including more samples and accounting for variables such as age, gender, immune-competency, co-morbidities and time post-infection would be invaluable in designing appropriate prevention and treatment strategies for these and other emerging infections.

## Concluding Remarks

Recent advances in cellular, molecular and computational techniques have provided detailed insights into the specificity and breadth of Ab responses. This in-depth knowledge has revealed the nuances of our immune system and its capacity to generate a pathogen-specific response, as described in this review.

Several considerations need to be taken into account when undertaking the arduous task of examining specific B cells at a single cell level. In addition, a successful transition toward clinical applications requires an understanding of the infecting pathogen or vaccine, the cell subset to be examined, the technology available and ultimately careful interpretation of the data.

Pathogens such as HIV and malaria lead to atypical memory, HIV and Influenza constantly adapt to evade the immune system, *S. pneumoniae* has several strains, *Dengue virus* worsens clinical disease upon reinfection and a primary infection with *C. difficile* renders the host prone to recurrent infections. Therefore, understanding the pathogen and its mechanism of action will allow better selection of antigenic targets to generate broadly neutralizing Ab.

The selection of the cell subset to be examined is crucial; specific Bmem are rare, but available months after the acute response has subsided and their Ab repertoire is reflective of a wider response providing a more “historical” perspective of the specific response. Plasmablasts peak shortly after vaccination or infection, do not require Ag-specific baiting for isolation and their repertoire is typically representative of the current response only. PCs are rare in the periphery but can be generated *in vitro* by Bmem differentiation and while they provide information on the existing memory, this methodology does not take into account the influence of the tissue microenvironment which cannot be fully recreated *ex vivo*.

While we have acquired considerable information of the Ag-specific B cell repertoire, we have just now begun to explore the possible existence of BCR “public lineages/clonotypes” in response to infection (177). Public clonotypes are described as the presence of dominant or nearly identical VDJ amino acid sequences across multiple individuals and have been better studied in the context of T cells rather than B cells. Analyzing Ig sequences shared across multiple individuals will be invaluable in understanding why these clonotypes emerge and the overall adaptive responses to specific pathogens.

In addition to infectious diseases, the analysis of B cell repertoires has improved our ability to detect immune system disorders and to elucidate the possible mechanisms causing them. For example, rheumatoid arthritis (RA) is a systemic autoimmune disease that often involves the production of anti-citrullinated protein Abs. Ab sequencing in RA patients has defined autoantibody specificities (178) and provided insights into clinical disease and the mechanisms leading to breaks in tolerance (179) and increased pro-inflammatory activity (180). In common variable immune deficiency, analysis of Ig H chain gene rearrangements demonstrated abnormal VDJ rearrangement and CDR3 formation, suggesting early differences in B cell selection and development in these patients (181). Finally, Ab studies have been pivotal in demonstrating the clonal origin of multiple myeloma (182) and more recently, sequencing technologies to define a patient's Ab repertoire have been applied clinically for disease detection and assessment of therapeutic response in myelomas (183) as well as leukemias (184).

Considering the knowledge and tools available to us, the goal of developing precise and personalized therapeutics is coming closer to fruition.

## Author Contributions

HS, KS, JW, CW, JB, RB, JJ, and ML contributed to the writing and editing of this review article.

### Conflict of Interest Statement

The authors declare that the research was conducted in the absence of any commercial or financial relationships that could be construed as a potential conflict of interest.
